# Un cas d'atrophie médullaire chez un plongeur sous-marin professionnel

**DOI:** 10.11604/pamj.2015.22.169.7908

**Published:** 2015-10-21

**Authors:** Samia Frioui, Sonia Jemni

**Affiliations:** 1 Service de Médecine Physique et de Réadaptation Fonctionnelle, CHU Sahloul Sousse, Faculté de Médecine « Ibn El Jazzar » Sousse, Tunisie

**Keywords:** Atrophie médullaire, plongée sous-marine, accident de décompression médullaire, paraplégie, IRM, Atrophie médullaire, plongée sous-marine, accident de décompression médullaire, paraplégie, IRM, medullary atrophy, scuba diving, medullary decompression accident, paraplegia, MRI

## Image en medicine

L'accident de décompression médullaire constitue la majorité des accidents de plongée (40 à 45%). Dans 20 à 30% des cas, les plongeurs conservent des séquelles neurologiques de gravité variable dont 10% de formes invalidantes. L'existence de signes déficitaires moteurs précoces, d'une aggravation des symptômes pendant le transport vers le centre hyperbare, et l'apparition de troubles sphinctériens sont des facteurs de mauvais pronostic. La symptomatologie des accidents de décompression médullaire est en rapport avec la survenue d'une ischémie aigue de la moelle épinière dont l'origine est imparfaitement comprise. Nous rapportons le cas d'un patient âgé de 34 ans, sans antécédents, plongeur professionnel, qui a présenté suite à un accident de plongée à 110 m, puis une remontée rapide une détresse respiratoire et une lourdeur des deux membres inférieurs. Il a été transporté dans un caisson hyperbare à l'hôpital. L'angioscanner réalisé en urgence a mis en évidence une embolie pulmonaire segmentaire antéro-basale droite. L'IRM cérébromédullaire a montré une lésion en hyper signal étendue de C3 à C6 d'allure ischémique. Après un an d’évolution, le patient garde toujours une paraplégie complète flasque ASIA A niveau D4 avec une anesthésie tactile et thermo-algésique, des réflexes ostéo-tendineux abolis, une hypotonie anale, des escarres (sacrée, trochantérienne gauche, ischiatique droite) stade III et une amyotrophie des deux membres inférieurs. Une IRM médullaire réalisée a montré une atrophie médullaire très importante étendue de D2 jusqu'au cône terminal (A, B, C, D). L’évolution d'un accident de plongée est imprévisible, avec un risque non négligeable de séquelles neurologiques.

**Figure 1:**
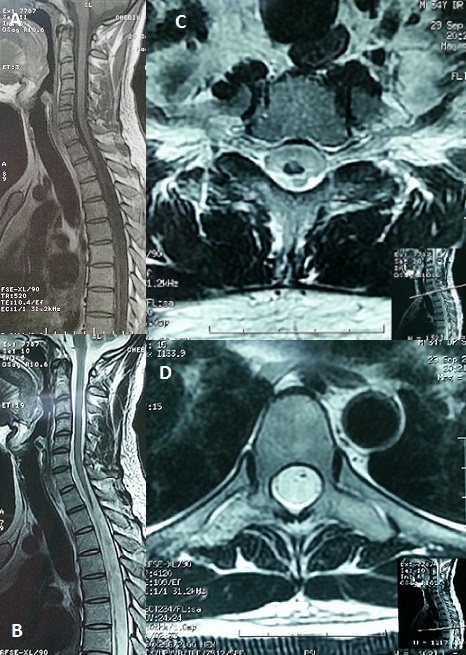
(A) IRM médullaire en coupe sagittale T1: une atrophie médullaire sévère; (B) IRM médullaire en coupe sagittale T2: atrophie médullaire sévère; (C) IRM médullaire en coupe axiale T2 niveau D1: atrophie médullaire importante; (D) IRM médullaire en coupe axiale T2 niveau D6: absence quasi-totale de la moelle

